# Adaptation and Evaluation of a Symptom-Monitoring Digital Health Intervention for Patients With Relapsed and Refractory Multiple Myeloma: Pilot Mixed-Methods Implementation Study

**DOI:** 10.2196/18982

**Published:** 2020-11-17

**Authors:** Noa Biran, Robin Anthony Kouyaté, Emre Yucel, Gillian E McGovern, Antoinette M Schoenthaler, Olivia G Durling, Rashmi Unawane, Andrew Schutt, Sumeet Panjabi

**Affiliations:** 1 Division of Multiple Myeloma John Theurer Cancer Center Hackensack University Medical Center Hackensack, NJ United States; 2 Amgen Inc Thousand Oaks, CA United States; 3 Rip Road Inc New York, NY United States; 4 Department of Population Health NYU School of Medicine New York, NY United States; 5 Medocity Inc Parsippany, NJ United States

**Keywords:** mHealth, digital health, electronic patient-reported outcome, ePRO, patient-reported outcome, PRO, mobile, app, implementation science, multiple myeloma, relapsed refractory multiple myeloma

## Abstract

**Background:**

Relapsed and refractory multiple myeloma (RRMM) is a bone marrow cancer that requires systemic treatment, which often results in severe symptom burden. Recent studies have found that electronic patient-reported outcome (ePRO) interventions implemented in the clinic setting have had positive outcomes for other oncology populations. Evidence of the efficacy of a similar approach is lacking for patients with RRMM.

**Objective:**

Recent recommendations for digital health interventions call for the publication of descriptions of iterative development processes in order to improve reproducibility and comparability. This study is an implementation pilot aiming to evaluate the acceptability and appropriateness of an ePRO intervention for patients with RRMM and to explore its impact on clinic workflow.

**Methods:**

A total of 11 patients with RRMM were recruited from the John Theurer Cancer Center in Hackensack, New Jersey. Patients used a mobile app to report on 17 symptoms at 4 sessions, each a week apart. Patients could also report symptoms ad hoc. When reports met predefined thresholds, the clinic was alerted and patients received automated guidance. Study end points were assessed using qualitative and quantitative methods.

**Results:**

A total of 9 patients (mean age 69.7 years) completed the study. Overall, 83% (30/36) of weekly sessions were completed. Patients found the frequency and time required to complete reporting acceptable. All patients agreed that the app was easy to use and understand. Providers felt the alerts they received required refinement. Patients and providers agreed it would be beneficial for patients to report for longer than 4 weeks. Patients felt that the training they received was adequate but contained too much information for a single session. All patients found the symptoms tracked to be appropriate; providers suggested shortening the list. All patients understood how to use the app for weekly reporting but had confusion about using it ad hoc. Providers felt the ad hoc feature could be removed. Neither patients nor providers viewed the in-app data reports but agreed on their potential value. Patients reported benefitting from symptom reporting through increased awareness of their symptoms. Clinic staff reported that app alerts were too numerous and redundant. They had difficulty responding to alerts within their existing workflow, partially because the data were not integrated into the electronic medical record system.

**Conclusions:**

Overall, the intervention was found to be acceptable and appropriate for patients with RRMM. Points of friction integrating the intervention into the clinic workflow were identified. Clinic staff provided recommendations for addressing these issues. Once such modifications are implemented, ePRO data from patients with RRMM could be used to inform and improve clinical research and care. This study underlines the importance of an iterative approach to implementation that includes all stakeholders in order to ensure successful adoption.

## Introduction

Multiple myeloma (MM) is an incurable hematological neoplastic disorder characterized by uncontrolled proliferation of clonal plasma cells (ie, myeloma cells) in the bone marrow [1,2]. Nearly all patients who have MM eventually relapse or become refractory to treatment, which is known as relapsed and refractory multiple myeloma (RRMM) [3]. Patients remain on systemic chemotherapy for the entirety of their disease course and the rest of their life following diagnosis. Many of these cancer therapies carry substantial toxicity burdens [4]. For this reason, the primary goals of treatment are to extend survival while maintaining or improving the patient’s quality of life, provide lasting relief from disease- and treatment-related symptoms, obtain maximum benefit from treatment, and manage remission [3,5].

Recently, there has been a focused effort to assess the patient experience in health care via patient-reported outcomes (PROs) [6,7]. A PRO is any report of the status of a patient’s health condition that comes directly from the patient without interpretation of the patient’s response by a clinician or any other third party [8]. PRO measures are often self-completed questionnaires that can be used to capture data on various domains, including functional status, health-related quality of life, symptom burden, treatment experience, emotional well-being, and health-related behaviors [6,9-12].

Studies in oncology have explored how to effectively implement PRO interventions in the cancer care setting [13-17] and assessed their impact on patient-centered outcomes, health outcomes, and overall survival [18-20]. Electronic PROs (ePROs), which are collected using electronic formats, are preferable, as they allow for systematically timed reporting between clinic visits, automated reminders to complete reporting, automated alerts to investigators, and real-time monitoring of compliance [7]. Approaches to systematic collection of data using ePRO systems have been shown to prompt clinical action for symptom management [21] and make care more patient centered [22]. One study, conducted at Memorial Sloan Kettering Cancer Center among patients initiating chemotherapy for certain metastatic solid tumors, found that patients using an ePRO system had greater improvement in health-related quality of life, were less frequently admitted to the emergency department or hospital, remained on chemotherapy longer, and had increased survival compared with patients receiving usual care (ie, discussion and documentation of symptoms during clinic visits, with patient encouragement to call about concerning symptoms between visits) [18,19]. To our knowledge, evidence for the efficacy of a similar approach is lacking for patients with RRMM.

Recent recommendations for digital health interventions call for the publication of detailed and transparent descriptions of iterative development processes in order to improve the reproducibility and comparability of digital health interventions in research (eg, randomized controlled trials) and in clinical practice settings [23-25]. This is the basis of implementation science, which is focused on “understanding and accelerating the integration of research findings and research-based innovations into everyday practice settings to improve health” [26]. Multiple frameworks to inform these iterative processes have been published to guide researchers [24,27], including the mobile health (mHealth) Development and Evaluation framework [25], which guided this study. The mHealth Development and Evaluation framework outlines several phases, including focus groups, pretesting, and pilot testing with a small sample from the target audience, in order to ensure the intervention is engaging and useful to the target users before use in randomized controlled trials [25].

This study was a content pretest and implementation pilot intended to assess the adaptation of a research-based ePRO intervention for use among patients with RRMM in their oncology care setting. The study was designed to collect feedback from patients and their providers regarding the acceptability and appropriateness of the intervention and the content included within it. The results are intended to inform and provide guidance for future iterations of ePRO initiatives delivered in a clinic setting for this or a similar patient population.

## Methods

### Study Sample

Starting January 2019, a purposive sampling strategy was used to recruit patients with RRMM receiving care at the John Theurer Cancer Center at Hackensack University Medical Center in Hackensack, New Jersey. Based on the study design, objectives, and existing research, information power was assessed to be relatively high and, as such, the desired sample size was determined to be approximately 10 patients [28]. Potential study participants were identified through review of the clinic’s electronic medical record (EMR) system. Identified patients were approached by their treating oncologist at their next clinic visit to assess interest and confirm eligibility. All participants provided written informed consent prior to participation. Clinicians who were involved in the treatment of the recruited patients also participated in the study. The clinical team included a lead hematology oncologist, a nurse practitioner, a nurse clinician, and a research assistant. The institutional review board at the Hackensack University Medical Center approved this study.

The following patient inclusion criteria were used: (1) age of 18 years or older at the time of enrollment; (2) diagnosis of RRMM; (3) initiation of or active treatment with second, third, or fourth line of therapy; (4) treatment that was expected to continue for at least four weeks from the time of enrollment; and (5) treatment that took place at John Theurer Cancer Center. Patients were excluded if they met any of the following criteria: (1) current participation in an investigational treatment, (2) Eastern Cooperative Oncology Group Scale of Performance Status score greater than 2, (3) the inability to read or comprehend English, (4) lack of internet access at their place of residence, (5) inability to receive email or text messages, or (6) refusal to provide informed consent.

### Intervention Overview

**Figure 1:**
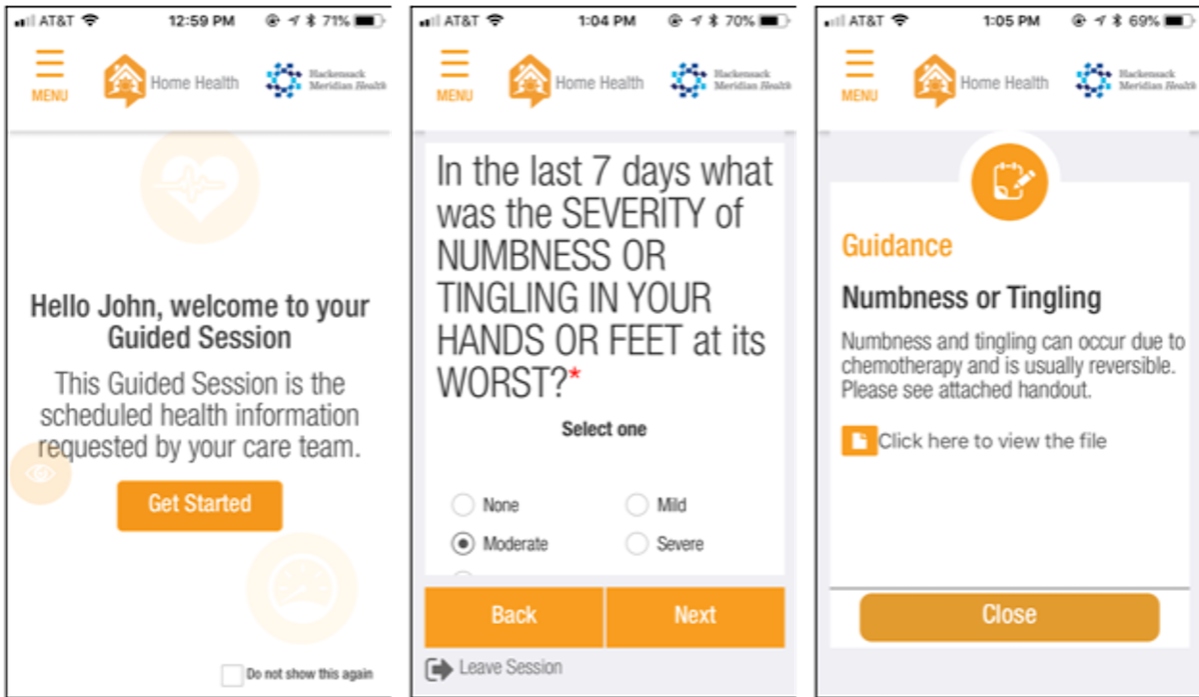
Screenshots of the Medocity Home Health app.

Before patient recruitment, a cocreation process including the clinic staff, technology provider, and research team was conducted to adapt and refine the intervention [18] for use with patients with RRMM and determine how to integrate it into the John Theurer Cancer Center clinic setting. The result of the working sessions was the intervention protocol and an iteration of the Medocity Home Health app to be used for ePRO collection (Figure 1). App use was governed by Medocity’s privacy policy [29].

Patients were trained by the research assistant on the intervention protocol and use of the app immediately after study enrollment and collection of informed consent. Patients could access the app from the web or by downloading the native iOS or Android app from the app store on their personal mobile device. The app guided patients on what and when to report, visualized the reported data, and facilitated delivery of alerts and data reports to clinic staff.

Patients were asked to report 17 common RRMM PROs selected from the Patient-Reported Outcomes Common Terminology Criteria for Adverse Events (PRO-CTCAE) bank [30] (Table 1). The National Cancer Institute’s PRO-CTCAE was developed as a standard, patient-centered approach for accurately and reliably collecting symptomatic adverse events in oncology research [4]. PRO selection was based on expert clinical review and was led by the principal investigator. The goal of PRO selection was to create a parsimonious list that maximized clinical relevance and minimized burden and duplication for clinical practice [7].

**Table 1:** Selected Patient-Reported Outcomes Common Terminology Criteria for Adverse Events symptom list and alert logic.

Symptom	Original grade trigger	Revised grade trigger
Anxiety	3 & 4	Removed
Appetite loss	3 & 4	4
Constipation	3 & 4	4
Cough	3 & 4	4
Diarrhea	3 & 4	4
Fatigue	3 & 4	4
Fever	3	4
Headache	3 & 4	4
Insomnia	3 & 4	4
Nausea	3 & 4	4
Numbness or tingling	3 & 4	4
Pain	3 & 4	4
Rash	3	4
Sad feelings	3 & 4	Removed
Shortness of breath	2, 3, & 4	4
Swelling	3 & 4	4
Vomiting	3 & 4	4
Ad hoc^a^	2 & 3	Removed
Inactivity	1	1

^a^After an ad hoc symptom reporting session, patients are asked, “Would you like your healthcare team to be aware of an ongoing concern?” If the patient chose “no,” a grade 2 alert was created. If the patient chose “yes,” a grade 3 alert was created. There is no way to generate a grade 1 or grade 4 alert from ad hoc reporting.

Patients rated the severity, frequency, and interference (or a combination of 2 of the 3) for each PRO using a 5-point Likert scale. Severity grades ranged from “none” to “very severe,” frequency grades ranged from “never” to “almost constantly,” and interference grades ranged from “not at all” to “very much.” For rash and fever, patients were only asked about the symptom’s presence.

Ratings were used to derive a composite grade ranging from 1 to 4 (Table 2). If a rating met a specified grade, then an alert was generated (Table 1) for the provider and real-time self-management guidance was sent to the patient through the app. The self-management guidance contained information on practices patients could perform to address the relevant symptom. Patients were also encouraged to call the clinic or go to the emergency room if needed. Alerts were also generated based on patient inactivity, defined as not completing the weekly guided session within the first 24 hours after it became available. These alerts were classified as grade 1 and were not communicated to the clinicians.

Reporting was done during scheduled sessions, each occurring 1 week apart; these were known as weekly guided sessions. The first session was completed during study enrollment with the research assistant. Patients completed the remaining sessions on their own or with the assistance of a caregiver. Patients were sent a reminder at the same time each week through their choice of email, text message, or push notification when the next session was available. A session was only available for 48 hours starting from the time of the notification. Patients were not required to respond to all questions at once and could complete the session at any time within the 48-hour window. In addition to the scheduled sessions, patients could use the app to report any of the 17 symptoms ad hoc. Patients were instructed to use ad hoc reporting when they experienced symptoms between the reporting sessions so that the clinicians could actively monitor their symptoms. Patients we asked to complete 4 total weekly guided sessions.

**Table 2:** Composite symptom grade definitions.

Symptom grade	Severity	Frequency	Interference
Grade 1	Mild	Rarely	N/A^a^
Grade 2	Moderate	Occasionally	Somewhat
Grade 3	Severe	Frequently	Quite a bit
Grade 4	Very severe	Almost constantly	Very much

^a^N/A: not applicable.

### Clinic Workflow

At baseline, the clinic had 4 existing channels for patient communication: phone, email, the MyChart Patient Portal, and an online forum for patients to ask nonurgent questions. Clinic protocol required that all incoming patient communication be responded to by the end of the business day. Communications marked as urgent had to be responded to within one hour.

The staff members had access to a clinician version of the ePRO app (also available as a web portal and native mobile app), in which they could view and respond to reported symptoms and manage alerts. During the initial working sessions, stakeholders decided not to integrate app data into the clinic’s EMR system until concept pretesting and refinement was complete. In order to reduce the time it took clinicians to become aware of app alerts, the research assistant manually transferred alert data from the clinician app into the clinic’s EMR system and communicated to the appropriate clinician that there were new data to review. The ePRO app was considered a fifth channel of communication by the clinic and, as such, alerts were responded to by the end of the business day.

**Figure 2:**
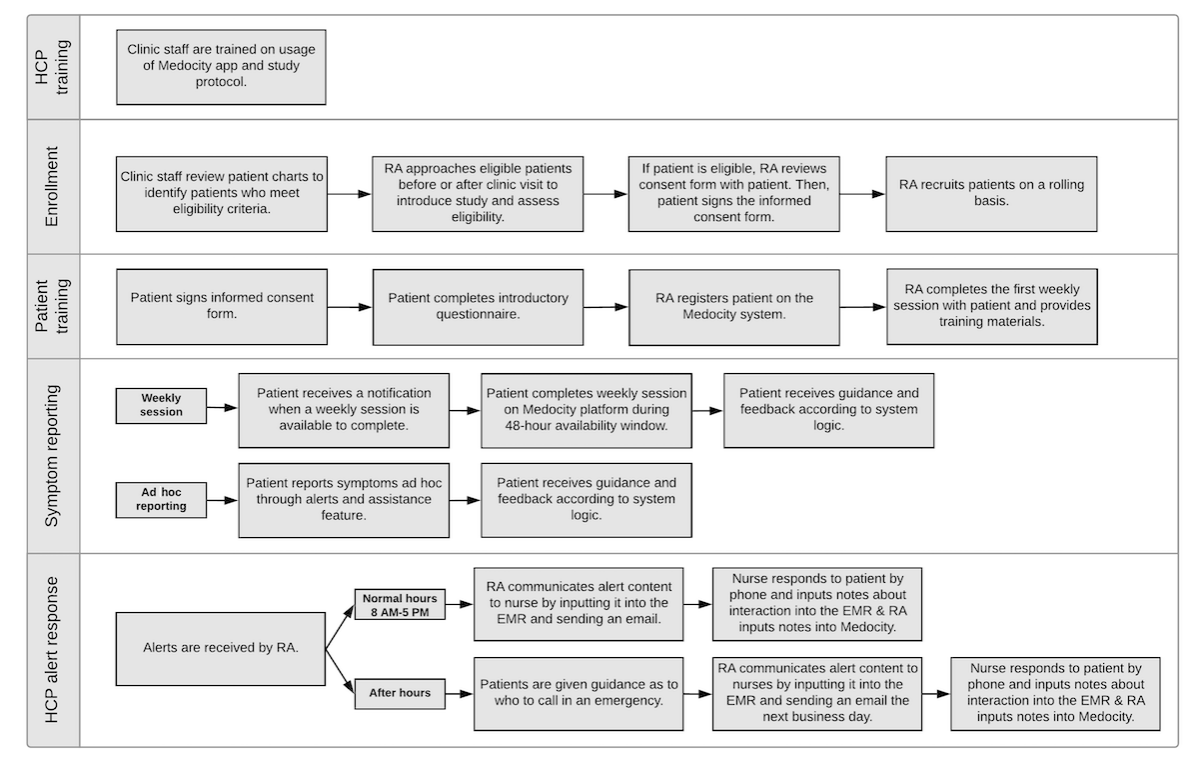
Planned study workflow. EMR: electronic medical record; HCP: health care professional; RA: research assistant.

Participating staff members were trained on the intervention protocol and use of the app prior to study onset. Clinicians could incorporate the PRO data into routine care; however, no protocol was defined for how clinicians should use the data. The workflow as it was planned to be executed during the study is visualized in Figure 2.

### Operationalization of Implementation Outcomes

Acceptability is defined as the perception among stakeholders that an intervention or innovation is agreeable [31]. It is a critical factor to assess when planning an intervention, as unacceptable tools will likely have low usage in the target population [32]. For the purpose of this study, acceptability was operationalized through the following end points derived from interviews and app usage data: completion of the weekly guided sessions and ad hoc reports*,* frequency of reporting, clarity of the app content, perceived ease of use, amount of time required to complete reporting, reporting duration, and clinic response to reported symptoms.

Appropriateness is defined as the “perceived fit, relevance, or compatibility of the innovation or evidence-based practice for a given practice setting, provider, or consumer; and/or perceived fit of the innovation to address a particular issue or problem” [32]. This construct can help elucidate whether an intervention will be effective in the intended environment and is suitable for the target population. Appropriateness was operationalized through the following end points derived from qualitative interviews: adequacy of the training, relevance of the 17 preselected PRO symptoms to patients with RRMM, patient comprehension of the use of the app for structured and ad hoc reporting, sensitivity of the logic that triggers alert notifications, frequency and volume of the app alerts, utility of the graphs and reports, effect on the patient’s perceptions of their oncology care, and fit within existing clinic workflow.

### Data Collection Methods

It is recommended for implementation research that a mix of qualitative and quantitative methods be used to explore and obtain an in-depth understanding not possible with one approach and data source alone [33]. The methods used to assess the implementation study outcomes were informed by the mHealth framework put forth by Peters et al [31] and Proctor et al [32].

The quantitative methods used included a paper-based patient enrollment questionnaire that assessed patients’ demographic information, technology use characteristics, and feelings towards their RRMM treatment and care (Multimedia Appendix 1); clinical data extracted from the EMR; app usage data; and 2 questions regarding perceived ease of app use and understandability of app content (adapted from Basch et al’s patient impressions exit questionnaire [13]), which were embedded into the qualitative interviews. The 2 questions were rated on a Likert scale ranging from 1 (strongly agree) to 4 (strongly disagree). Staff members were also given a paper-based questionnaire, administered approximately halfway through the study. They were asked about the perceived clarity of the alert content, the ease of responding to app alerts within their existing clinic workflow, and the utility of receiving alerts from patients between clinic visits (Multimedia Appendix 2).

The qualitative methods used included focus groups and individual interviews. Each patient participated in 2 semistructured individual interviews during the study: one 30-minute interview was conducted within two weeks of study initiation and one 1-hour interview was conducted within two weeks of study completion. The first interview used open-ended questions to assess patients’ initial thoughts, feelings, and expectations of the app. Patients were asked about their initial impressions of the app training, their experience with the weekly guided sessions, and their use of the ad hoc capability. One objective of this interview was early identification of any issues that may have hindered patients from participating.

The second interview explored patients’ thoughts about the app in more detail. Specifically, patients were asked open-ended questions to assess their opinions on the timing, frequency, and duration of PRO reporting via the weekly guided sessions; clarity of the app content; relevance of the reported symptoms; utility of the data reports; perceptions of the feedback received in response to reported symptoms; and perceptions of their cancer care before and after the intervention.

In order to support ad hoc PRO reporting, the standard 7-day PRO-CTCAE question recall period was modified to reference symptoms that occurred within the past 24 hours. A cognitive debriefing technique was used to assess comprehension of this adjusted scale during the second interview. Specifically, patients were shown an example ad hoc symptom question and asked to restate in their own words what the item meant in order to confirm their understanding [34].

Semistructured group and individual interviews were also conducted with the clinic staff at the end of the study period. Interview topics included the staff’s opinions of the frequency and duration of patient reporting, relevance of the selected symptoms, adequacy of the training on the intervention protocol, sensitivity of the logic that triggered app alerts, usefulness of the ad hoc reporting, utility of the graphs and reports, and fit of the intervention protocol within the clinic workflow. Impact on clinic workflow was further assessed through a workflow mapping of actual versus planned implementation.

### Data Analysis

An inductive analytic approach was used to analyze all qualitative data. This approach allows research findings to emerge from the frequent, dominant, or significant themes inherent in raw data without the restraints imposed by more structured methodologies [35]. All interviews were professionally transcribed, then reviewed by 3 independent reviewers with experience in qualitative methods. Themes from the interviews were extracted and categorized. After individual review, the transcripts were discussed as a group to ensure convergence of themes.

Analysis of the quantitative measures was limited to descriptive statistics. App usage data, patient demographics and clinical characteristics, and the staff experience questionnaire responses were analyzed as means, medians, and standard deviations for continuous variables and as frequencies and percentages for categorical variables where appropriate.

## Results

### Demographics and Clinical Characteristics

A total of 11 patients were approached for study inclusion and agreed to participate; 2 patients discontinued after the training session. One patient discontinued because they felt they were too busy to commit to the study and the other did not feel well enough to participate. Of the 9 patients who completed the study, the mean age was 69.7 (SD 6.5) years and 44% (4/9) were women (Table 3). Patients had been living with an MM diagnosis for a mean of 10.9 (SD 7.2) years and attending John Theurer Cancer Center for treatment for an average of 9.9 (SD 7.5) years. All patients included in the study had relapsed disease and required chronic therapy with frequent clinic visits: 33% (3/9) of patients were in their first relapse (second line of therapy) and 67% (6/9) were in their second relapse (third line of therapy).

**Table 3:** Patient demographic information and clinical characteristics.

Characteristic		Values
Age, mean (SD), range		69.67 (6.54), 56-76
Time since multiple myeloma diagnosis (years), mean (SD), range		10.89 (7.29), 2-23
Duration of treatment at HUMC^a^ (years), mean (SD), range		9.89 (7.46), 2-23
**Gender, n (%)**		
	Male	5 (56)
	Female	4 (44)
**Line of therapy, n (%)**		
	Second	3 (33)
	Third	6 (67)
**ECOG^b^ score at start of current therapy^c^, n**		
	0	1
	1	6
	2	1
**Highest education, n (%)**		
	Some high school, no diploma	1 (11)
	High school graduate/GED^d^	4 (44)
	Some college	1 (11)
	College graduate	1 (11)
	Some postgraduate	1 (11)
	Master's degree	1 (11)
**Household income^c^ (US $), n (%)**		
	25,000-34,999	1 (11)
	50,000-74,999	2 (22)
	75,000-99,999	2 (22)
	100,000-149,999	2 (22)
	150,000 or more	1 (11)
**Marital status, n (%)**		
	Single	3 (33)
	Married	5 (56)
	Widowed	1 (11)

^a^Hackensack University Medical Center.

^b^ECOG: Eastern Cooperative Oncology Group.

^c^1 patient declined to answer or data were unavailable.

^d^GED: General Educational Development.

### Acceptability

#### App Usage

There was an overall weekly guided session completion rate of 83% (30/36). The first weekly guided session, completed in the clinic during the training session, had a completion rate of 100% (9/9). Session 2 had a completion rate of 67% (6/9), session 3 had a completion rate of 89% (8/9), and session 4 had a completion rate of 78% (7/9). In total, 67% (6/9) of patients completed all 4 weekly guided sessions. There were 5 unique ad hoc reporting sessions completed by 5 separate patients, resulting in 23 total symptoms reported ad hoc.

#### Frequency and Timing of Reporting

The weekly guided sessions took an average of 4.5 minutes to complete. However, patients perceived them to take an average time of 19 minutes. All patients felt the time required to complete the weekly guided session was acceptable. All weekly guided sessions were completed on the same day that the reminder was received, except for 1, which was completed the day after.

During the interviews, patients agreed that reporting symptoms on a weekly basis was acceptable and not burdensome. All staff members felt that a weekly reporting schedule would be acceptable for patients who came to the clinic 1 to 2 times per month. However, they felt this would be too frequent for patients who came into the clinic more frequently. They felt that they were aware of the symptoms that frequently seen patients experienced and thus found the alerts from the app duplicative. They stated that there would be greater benefit from weekly reporting for patients who required less frequent visits because those patients had fewer points of contact with the clinic.

#### Clarity of Intervention Content and Perceived Ease of Use

All patients reported understanding the definition of each symptom and the scales used to report symptom severity and interference, including for ad hoc reporting. Patients responded favorably to the 2-item ease of use and app comprehension statements; all (9/9) patients strongly agreed with the statement “I found the Medocity Home Health app easy to use” at their exit interview. In addition, 78% (7/9) of patients strongly agreed and 22% (2/9) of patients agreed with the statement “Questions in the Medocity Home Health app were easy to understand.”

Staff members responded to the question “Please rate the clarity of the alert content that you received from patients enrolled in this study on a scale of 1 to 5 (1=not at all clear, 5=very clear).” One of 4 staff members responded with “3,” another responded “2,” and the remaining 2 staff members responded “1.”

#### Reporting Duration

Both patients and clinic staff agreed that it would be beneficial to report symptoms for longer than 4 weeks because it would allow them to see changes over time. The clinicians felt that longitudinal symptom data would help to improve clinical care by creating visualizations that put symptoms into perspective (eg, which symptoms were better, worse, new, or chronic). They also stated that consistently reported, longitudinal symptom data would help them make better decisions about how to manage long-term disease- and treatment-related symptom burden and help combat treatment fatigue, a psychological symptom associated with prolonged treatment engagement, which could increase duration on therapy and patient adherence to the clinical regimen [36].

#### Clinic Response to Reported Symptoms

A total of 33 symptom-related app alerts were generated over the study period. Clinicians reached out to patients in response to 76% (25/33) of the alerts of which they were notified. Of these, 15 alerts led to symptom counseling, 8 alerts resulted in clinicians advising patients to continue with a previously discussed management approach, 1 alert led to instructions to go to urgent care, and 1 alert led to instructions to go to urgent care and medication management. For the remaining 8 alerts, clinicians did not contact the patient because the reported symptom was chronic and already being treated.

Patients had mixed feelings about the clinic reaching out to them in response to the symptoms they reported in the app. Two patients mentioned that they appreciated having the clinic call them in response to their reporting because it reinforced that their data were being received. However, one patient reported that their symptoms did not warrant a phone call from the clinic due to its chronicity and low severity, and another patient expressed frustration at having to verbally repeat their symptoms after entering them in the app.

### Appropriateness

#### Adequacy of Training

Initial training on the app and intervention protocol took between 20 and 60 minutes per patient. No patient proposed modifications to the duration or timing of training completion. Patients felt equipped to use the app and record their symptoms following training.

Some patients reported feeling overwhelmed with the amount of information presented during the session and reported not remembering all topics that were discussed. While patients were given a handout that reinforced key information about the app and contained contact information for the research assistant, no patient recalled using the handout for reference or proactively reaching out to the research assistant with questions. However, some patients asked questions about the app and study when contacted by the research assistant for interview scheduling. All staff members agreed that training was comprehensive, but the patient session contained too much information to digest in one session.

#### Relevance of the Preselected Symptoms

Both patients and clinic staff felt that the 17 symptoms were relevant and appropriate for patients with RRMM. At the end of the study period, clinic staff suggested removing anxiety and sad feelings from the list of symptoms to maximize the clinical utility of the reports and reduce reporting burden on patients.

#### Comprehension of App Use

All patients reported understanding how and when to complete their structured PRO reporting; however, they did not report a clear understanding of how the reported data would be used in their care. Several patients thought the purpose of the intervention was to help other patients with RRMM through the use of aggregate data and did not realize their data could be used to help them directly. Patients did cite benefitting from participation through an increased awareness of their symptoms. This was achieved by taking moments of thoughtful reflection during the weekly guided sessions.

While all patients reported finding the ad hoc PRO reporting questions clear and easy to understand, some reported confusion regarding why and when it was appropriate to use the ad hoc reporting feature. Patients were unsure if they should report chronic symptoms or acute symptoms, if they should report symptoms related to their disease and treatment or general symptoms (eg, those related to the flu), and whether symptoms reported using the ad hoc feature should also be reported during the weekly guided session.

Two clinicians felt that ad hoc reporting was not appropriate for reporting emergency symptoms and mentioned that it may interfere with the clinic-established protocol for patients to call the clinic directly or go to the emergency room when they experience severe symptoms or that it may delay the process of a patient getting immediate help. However, another clinician felt that ad hoc reporting had the potential to catch emergency symptoms that the clinic might otherwise miss or that the patient might identify as a nonemergency and stated that earlier awareness of these emergency symptoms could prevent the need for a more aggressive intervention.

#### Utility of Symptom Graphs

Most patients were not aware that symptom graphs were available in the app or did not view them after initially seeing them during the app training session. When patients were shown example PRO reports in the interviews, they felt the graphs could be helpful if they showed data over a longer period of time or showed significant fluctuations in symptoms over time.

The clinicians reported not using symptom graphs and reports during the study due to the high volume of information that typically needs to be reviewed before and during a clinic visit and the lack of integration of the data into the EMR system and routine visit workflow. They did see the potential value in a summary of longitudinal PRO data because it could allow them to address patient concerns proactively and increase the efficiency and quality of a clinic visit through improved data transparency and shared knowledge.

#### Appropriateness of Alerts

**Figure 3:**
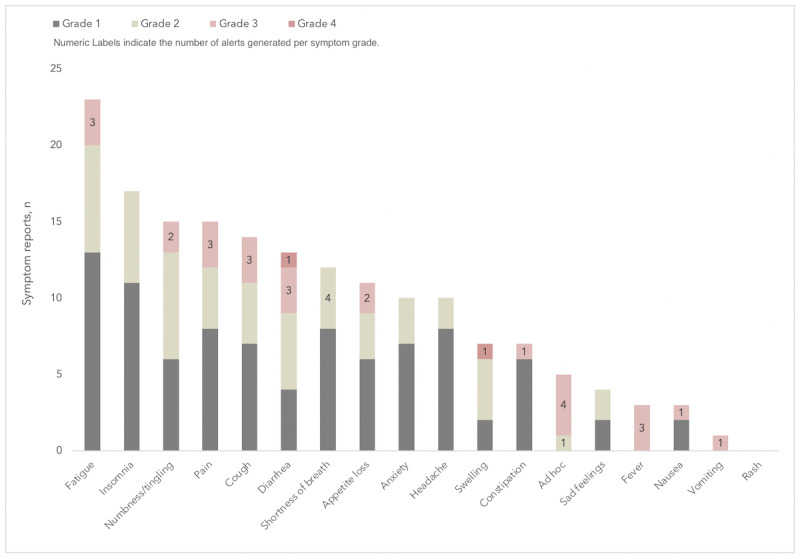
Frequency of reported symptoms and alerts.

A total of 62 alerts were generated over the course of the study. Of these 62 alerts, 29 (47%) were rated as grade 1 (inactivity) alerts and the remaining 33 (53%) were symptom related. Of the 33 symptom-related alerts, 15% (5/33) were grade 2 alerts, 79% (26/33) were grade 3 alerts, and 6% (2/33) were grade 4 alerts. Ad hoc reports (n=5) triggered the most alerts, followed by diarrhea (n=4) and shortness of breath (n=4) (Figure 3).

Clinicians, particularly the 2 nurses, reported that the volume of alerts generated was too high, alerts did not always map to clinically relevant criteria, and alerts tended to be redundant, with symptoms that had already been addressed through the clinic’s established communication channels. When responding to the question “How helpful do you think it is to receive symptom alerts from patients between clinic visits? (Scale: 1=not at all helpful, 5=very helpful),” 3 of 4 staff members responded “1,” and 1 staff member responded “2.” Staff members explained that it would be most useful to be alerted of newly emerging, severe, or worsening symptoms; however, the alert logic also frequently captured chronic and expected symptoms. In order to maximize clinical utility, they suggested the alert criteria be made more specific so that more actionable app alerts were generated (Table 1).

#### Effect on Clinic Workflow

Overall, the clinic staff did not feel the current implementation of the intervention was easy to incorporate into their existing workflow. When asked, “How easy is it to respond to the intervention alerts as part of your regular clinic workflow? (Scale: 1=Not at all easy to 5=Very easy),” 3 of 4 staff members responded “1,” and 1 responded “2.” When probed, staff members explained that integrating the intervention alerts into their existing communication channels would greatly increase the usability.

#### Influence on Oncology Care

Patients reported a high level of satisfaction with their RRMM care during both qualitative interviews. During the second interview, patients were shown abstract emotional imagery and asked to select an image that best represented their relationship with their providers. All patients selected images that evoked positive qualities and described themes of partnership, equality, and teamwork when asked to describe how the image made them feel (Multimedia Appendix 3).

Patients did not feel that participating in the intervention impacted their relationship with their providers. This was likely due to very high baseline relationship quality, the short study duration, the lack of data integration into clinic visits, and the benefits of the intervention being entangled with the benefits of routine care.

During the clinic staff group interview, staff theorized that an ePRO intervention could have the following benefits for patients: (1) encouragement to remain on therapy as a result of more effective symptom management; (2) more control over their symptoms through increased symptom awareness, recognition of symptom patterns, and easily accessible self-management guidance; (3) an improved relationship with the clinical team due to consistent communication through a trustworthy platform; and (4) empowerment in clinic visits through the use of PRO reports as communication tools.

## Discussion

### Principal Results

This study attempted to redefine the protocol for an ePRO intervention, adapted from the protocol used by Basch et al [18], for use with patients with RRMM receiving care at the John Theurer Cancer Center. Overall, the intervention was found to be acceptable and appropriate for patients with RRMM. There was a high completion rate of the weekly reporting sessions. Patients understood how and when to use the app and did not find the reporting frequency burdensome, although staff members suggested weekly reporting may be more beneficial for patients who have less frequent clinic visits. Both patients and clinic staff agreed that it would be beneficial to report symptoms for longer than 4 weeks due to the nature and progression of RRMM. All felt that the 17 selected symptoms were relevant to patients with RRMM, but staff members suggested removing questions related to mental health symptoms to maximize clinical utility and reduce reporting burden. No impact was found on the patient-provider relationship, but this was likely due to the short intervention duration, lack of integration into routine practice, and the high-quality relationship at baseline. Clinicians confirmed that such an intervention could be effectively implemented into their clinic workflow; however, modifications are required, primarily with app alerts. Challenges were observed due to app alerts adding to clinician burden, alerts being unable to differentiate between chronic and acute symptoms, patient misunderstanding of when to use ad hoc reporting, and lack of integration of the intervention data into routine care.

Each medical practice has unique qualities, strengths, and limitations. The design and development of an ePRO intervention for use in the clinic setting should take these factors into consideration and include the input of the end users along the way in order to create a program that can be successfully adopted and executed [37]. Integrating new protocols into an existing clinical workflow requires an iterative process, and the first step is to ensure the protocol is well defined in order to be usable in practice [27].

The “Recommendations for Future Implementation” section describes 5 recommendations for improving this intervention for use at the John Theurer Cancer Center and other clinics with similar characteristics. Not all recommendations will be relevant to every clinic setting, but many findings may be transferrable, and the detailed and iterative approach adopted herein can be used as an example of implementation science in mHealth research.

### Recommendations for Future Implementation

First, patient training should be reduced in scope or broken into multiple sessions. Additional materials could be distributed electronically or at clinic visits to reinforce core concepts. Training should include content related to the purpose of PRO reporting and its intended use in the patient’s care regimen. Setting expectations for the patient and ensuring their understanding will help them be active participants in the intervention.

Second, the symptom list and alert logic should be designed to limit the overall volume of alerts and maximize their clinical relevance. This will reduce the burden on clinic workflow, limit the burden of patient reporting, and create a data set of the most relevant symptom information for use in clinical care. It is essential to differentiate acute versus chronic versus acute-on-chronic symptoms. One approach to increasing the relevance of app alerts is to triage them by including follow-up questions within the app. Follow-up questions could clarify whether the symptom has already been discussed or whether it is new or worsening, allowing the clinical team to triage the symptom’s urgency and plan patient outreach (Multimedia Appendix 4). Other authors have suggested assessing patients’ baseline symptom burden and interpreting their PRO reports accordingly [38].

Third, clinic staff suggested removing the ad hoc reporting feature in favor of relying on the clinic’s existing communication channels to capture urgent symptoms. This was proposed to reduce patient confusion, limit the total volume of alerts, minimize redundancy of reported symptoms, and reinforce the purpose of weekly reporting. This feature or a similar version could be retained for clinics with less robust patient communication channels.

Fourth, the ePRO reporting mechanism should be integrated into the clinic’s workflow. For example, intervention data could be integrated into the clinic’s EMR system or other existing patient content (eg, paper-based calendars). Communication flows should be clearly established, and points of friction should be identified and eliminated at the beginning of the intervention implementation to facilitate successful adoption.

Fifth, protocols should ensure that PRO data are integrated into clinic visits. For example, staff members said it would have been helpful to include a printed graph of active symptoms in front of the patient chart prior to each visit. Another strategy is having clinicians set patient expectations for how the PRO information will be used in their care and encouraging patients to review their data between clinic visits.

### Limitations

Some study limitations should be considered. First, this was a pilot study that included a small sample of patients with RRMM and clinic staff located at one study site, which may limit the generalizability of the findings. The 4 staff members came from different specialties, further limiting the generalizability of the staff member feedback. However, the feedback provided was valuable in creating preliminary insights from a range of providers involved in the symptom-reporting clinic workflow. Additional clinicians from each field should be included in subsequent implementations in order to gather a more representative perspective. Second, because the study was a pretest, it had a short duration of only 4 weeks. Future studies should be longer in order to investigate the impact that longitudinal symptom data can have on patient-clinician interactions and symptom management. Third, the study participants had been living with RRMM for many years and their disease was well controlled. As such, these findings cannot be applied to patients who are newly diagnosed with MM or patients with more acute symptom profiles. Fourth, this population had strong relationships with the clinical team prior to the study. This limited the ability to assess how participating in the intervention could improve the patient-provider relationship. Finally, interrater reliability was not calculated for the qualitative analysis due to the methodology employed. The themes identified in patient and clinician interviews should be validated in future work.

### Conclusions

The recent push to integrate patients’ voices into oncology care highlights the importance of identifying and addressing barriers to implementing PROs in clinical practice [26,39]. In addition, some have argued that iterative user-centered design strategies can be complementary to implementation science strategies in bridging “the research to practice gap” [40] by supporting the iterative refinement of interventions when translating to new populations or settings in order to systematically maximize “intervention-setting fit” [41]. We found this approach particularly important when adapting this intervention to address the unique aspects of a different population and clinic setting.

This implementation pilot study demonstrates how a successful ePRO intervention for patients with solid tumors could be adapted from research into the clinical setting for another patient population. This study underlines the importance of a systematic and iterative approach to implementation that includes all stakeholders in order to ensure successful adoption. Future research should consider these findings when attempting effective implementation of ePRO interventions in various oncology care practice settings.

## Appendix

Multimedia Appendix 1 Patient enrollment questionnaire.

Multimedia Appendix 2 Provider questionnaire.

Multimedia Appendix 3 Emotional imagery.

Multimedia Appendix 4 Proposed follow-up questions.

## Abbreviations

EMR: electronic medical record
ePRO: electronic patient-reported outcome
mHealth: mobile health
MM: multiple myeloma
PRO: patient-reported outcome
PRO-CTCAE: Patient-Reported Outcomes Common Terminology Criteria for Adverse Events
RRMM: relapsed and refractory multiple myeloma
