# Supplementary material for: Adaptation and Evaluation of a Symptom-Monitoring Digital Health Intervention for Patients With Relapsed and Refractory Multiple Myeloma: Pilot Mixed-Methods Implementation Study
Source: JMIR Form Res. 2020 Nov 17;4(11):e18982. doi: 10.2196/18982 (PMC7709004; doi:10.2196/18982)
Supplement: Multimedia Appendix 1 [file formative_v4i11e18982_app1.pdf]

This is a Multimedia Appendix to a full manuscript published in the J Med Internet Res. For full copyright and citation information see http://dx.doi.org/10.2196/jmir.18982

Multiple Myeloma ePRO Study Introductory Questionnaire

1. What is the highest level of education that you have completed? (Select one)

   - ☐ 8th grade or less
   - ☐ Some high school, no diploma
   - ☐ High school graduate, diploma or the equivalent (e.g. GED)
   - ☐ Some college, no degree
   - ☐ Trade/technical/vocational training
   - ☐ College graduate, associate or bachelor's degree
   - ☐ Some postgraduate, no degree
   - ☐ Master's degree
   - ☐ Doctorate degree

2. What was your total annual household income last year before taxes? (Select one)

   - ☐ Less than $25,000
   - ☐ $25,000 - $34,999
   - ☐ $35,000 - $49,999
   - ☐ $50,000 - $74,999
   - ☐ $75,000 - $99,999
   - ☐ $100,000 - $149,999
   - ☐ $150,000 or more
   - ☐ I prefer not to answer

3. What is your current marital status? (Select one)

   ☐ Single ☐ Married

4. How many individuals are in your household? (Enter a number; if you live alone, enter '1')

   # of individuals _____

5. Who do you live with? (Select all that apply)

   - ☐ My spouse/partner
   - ☐ Child/children (< 18 years)
   - ☐ Adult child/children (≥ 18 years)
   - ☐ Parent(s)
   - ☐ Other adult(s)
   - ☐ Nobody, I live alone

6. Are you the primary caregiver for any of the people in question 8? (Select all that apply)

   ☐ My spouse/partner
   ☐ Child/children (< 18 years)
   ☐ Adult child/children (≥ 18 years)
   ☐ Parent(s)
   ☐ Other adult(s)
   ☐ None or Not Applicable

7. Do you use a mobile device (smart phone, tablet, etc.)? (Select one)

   ☐ Yes ☐ No

8. In the past week, which of the following activities have you done with your mobile device? (Select all that apply)

   ☐ Check email
   ☐ Browse the internet
   ☐ Post to social media (e.g. Facebook, Twitter, Instragram, etc.)
   ☐ Download a new app
   ☐ Install a new app
   ☐ Use the calendar app to book or keep an appointment
   ☐ Set an alarm or timer
   ☐ Used an app for a health related purpose (track weight, steps, physical activity, etc.)
   ☐ Listen to music (e.g. Spotify, Pandora, iTunes, etc.)
   ☐ Book travel arrangements
   ☐ Watch a movie/show (e.g. Netflix, Hulu, HBOGo, etc.)
   ☐ Book a car ride share (e.g. Uber, Lyft, etc.)
   ☐ Listen and/or download a podcast
   ☐ Navigate directions (e.g. Apple Maps, Google Maps, etc.)
   ☐ Check stocks
   ☐ Connect to a Bluetooth device (headphones, car, speaker, etc.)
   ☐ Use the phone’s personal hotspot to connect another device
   ☐ Transfer photos or files via AirDrop

9. Please rate the following statements. (Circle one number per row)

	1<br>Strongly Agree	2<br>Agree	3<br>Somewh at Agree	4<br>Neither Agree or Disagree	5<br>Somewh at Disagree	6<br>Disagree	7<br>Strongly Disagree
I am usually among the first to try new technological devices	1	2	3	4	5	6	7
I get a kick out of buying new high-tech items before most other people even know they exist	1	2	3	4	5	6	7
I get a thrill out of being first to purchase a high-tech item	1	2	3	4	5	6	7
I consider myself to be a risk taker when it comes to new high-tech products.	1	2	3	4	5	6	7
When dealing with high-tech gadgets, I am usually one of the first in line to buy	1	2	3	4	5	6	7
I often try new technological devices before my friends and neighbors do	1	2	3	4	5	6	7
I am usually ahead of the average person when it comes to buying new high-tech products	1	2	3	4	5	6	7
Although I might not be the first to own innovative high tech devices, I usually get them before most people do	1	2	3	4	5	6	7
I consider myself to be a little more innovative than the average person when it comes to trying out new technological devices and services	1	2	3	4	5	6	7

10. Which of the following statements best describes how you feel about treatment and management of Multiple Myeloma? (Select one)

- ☐ I take charge of my care and pursue all the best options
- ☐ I'll do what I have to in order to get things back to normal
- ☐ It's out of my hands; I just have to be hopeful
- ☐ I'll do my part, but otherwise, I try not to think too much about it

11. Which of the following statements best describes how you feel about treatment and management of Multiple Myeloma? (Select one)

- ☐ I listen to my doctor's recommendations, but make the final decision myself after doing my own research
- ☐ I always follow my doctor's advice as s/he knows best

12. Which of the following statements best describes how you feel about treatment and management of Multiple Myeloma? (Select one)

- ☐ I usually feel in control when making decisions pertaining to my care
- ☐ Many times I feel that I don't have much control over decisions pertaining to my care

13. Which of the following statements best describes how you feel about treatment and management of Multiple Myeloma? (Select one)

- ☐ I enjoy connecting with others with Multiple Myeloma, even apart from support groups
- ☐ I prefer not to talk much about my Multiple Myeloma

14. Which of the following statements best describes how you feel about treatment and management of Multiple Myeloma? (Select one)

- ☐ I consider all the aspects of a treatment when considering whether to go on it
- ☐ I base treatment decisions primarily on whether it is an effective treatment that my doctor recommends
