# Supplementary material for: Adaptation and Evaluation of a Symptom-Monitoring Digital Health Intervention for Patients With Relapsed and Refractory Multiple Myeloma: Pilot Mixed-Methods Implementation Study
Source: JMIR Form Res. 2020 Nov 17;4(11):e18982. doi: 10.2196/18982 (PMC7709004; doi:10.2196/18982)
Supplement: Multimedia Appendix 2 [file formative_v4i11e18982_app2.pdf]

This is a Multimedia Appendix to a full manuscript published in the J Med Internet Res. For full copyright and citation information see http://dx.doi.org/10.2196/jmir.18982

Completed By:
Date:

Multiple Myeloma ePRO Implementation Study - Check-In Survey

This survey is to assess your engagement with the Medocity platform during the first few weeks of the ePRO MM study. Please take 5-10 minutes to answer this survey so we can make enhancements and improvements to the platform.

1. Since the study began, have you received any alerts through the EMR from patients that were utilizing the Medocity Home Health app?

   Yes   No   Not sure

2. If yes, please rate the clarity of the alert content that you received from patients enrolled in this study on a scale of 1 to 5. (1 = not at all clear, 5 = very clear)

   1   2   3   4   5

3. Did you reach out to a patient because of an EMR alert that was generated by a patient using the Medocity Home Health app?

   Yes   No   Not sure

4. How easy is it to respond to the EMR alerts as part of your regular clinic workflow? (1 = not at all easy, 5 = very easy)

   1   2   3   4   5

5. How helpful do you think it is to receive symptom alerts from patients in-between clinic visit in this ? (1 = not at all helpful, 5 = very helpful)

   1   2   3   4   5

6. Now that you've used the system for some time, what suggestions or comments do you have to improve it, or its implementation at the clinic?
