# Supplementary figures and images for: Adaptation and Evaluation of a Symptom-Monitoring Digital Health Intervention for Patients With Relapsed and Refractory Multiple Myeloma: Pilot Mixed-Methods Implementation Study
Source: JMIR Form Res. 2020 Nov 17;4(11):e18982. doi: 10.2196/18982 (PMC7709004; doi:10.2196/18982)

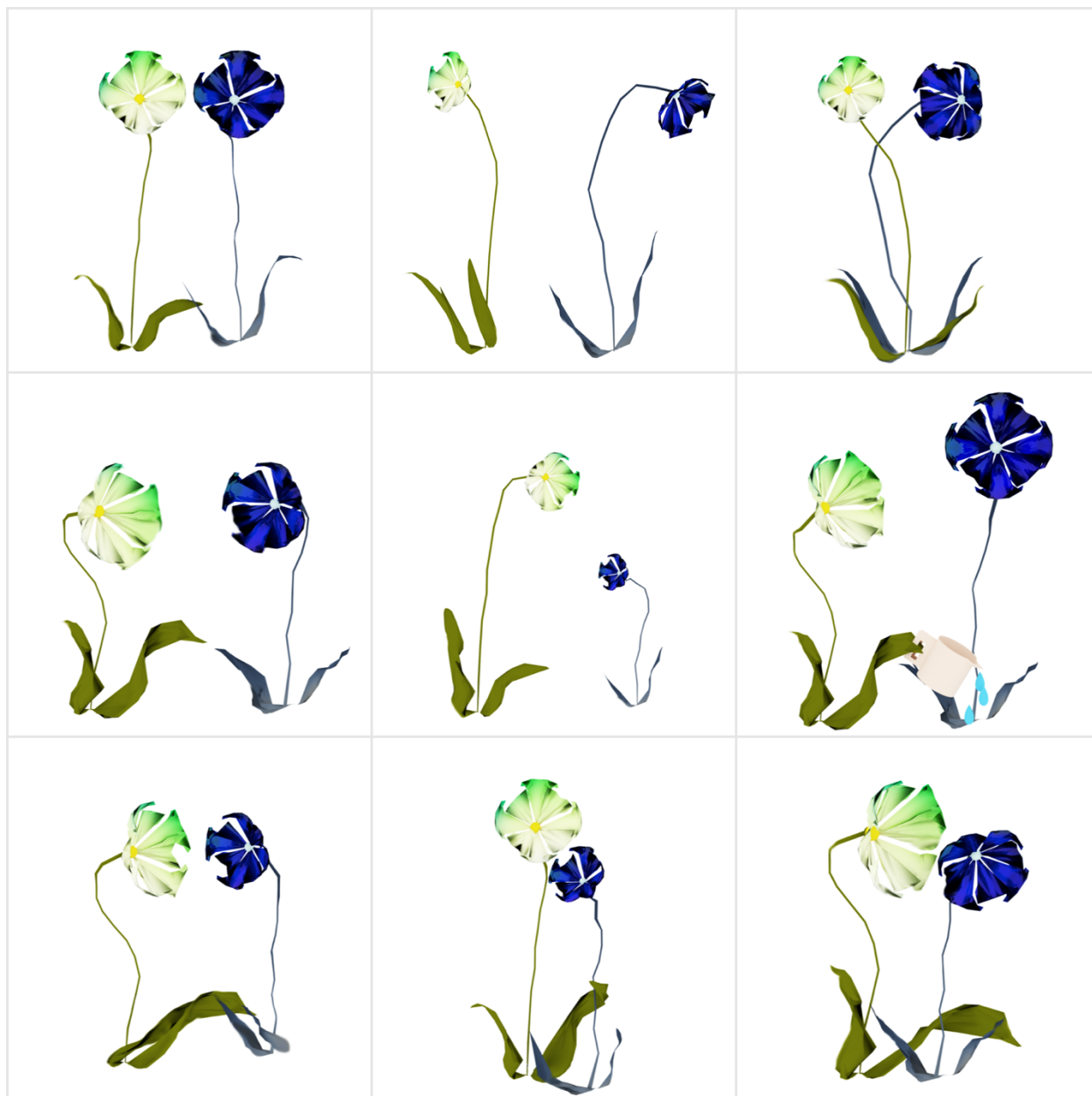

Supplement: Multimedia Appendix 3 [file formative_v4i11e18982_app3.pdf]
