# Supplementary material for: Adaptation and Evaluation of a Symptom-Monitoring Digital Health Intervention for Patients With Relapsed and Refractory Multiple Myeloma: Pilot Mixed-Methods Implementation Study
Source: JMIR Form Res. 2020 Nov 17;4(11):e18982. doi: 10.2196/18982 (PMC7709004; doi:10.2196/18982)
Supplement: Multimedia Appendix 4 [file formative_v4i11e18982_app4.pdf]

Added Follow-up Questions

To identify if symptom previously discussed*

Pain

Q1: **In the last 7 days, how OFTEN did you feel PAIN?**

A) Never

B) Rarely → Q1.B: **In the last 7 days, what was the SEVERITY of your PAIN at its WORST?**

- a) None
- b) Mild → Q1.B.b: **In the last 7 days, how much did PAIN INTERFERE with your usual or daily activities?**
  Not at all / A little bit / Somewhat / Quite a bit / Very much
- c) Moderate → Q1.B.c1: **In the last 7 days, how much did PAIN INTERFERE with your usual or daily activities?**
  Not at all / A little bit / Somewhat / Quite a bit / Very much

  Q1.B.c2: **Is/was this symptom new or worsening?**
  - Yes → Q1.B.c2.1: **Have you discussed this change with your clinical team?**
    - Yes
    - No
  - No

*With these added questions, removed Ad Hoc reporting

To make alerts more clinically meaningful*

Shortness of Breath (Dyspnea)

Q1: **In the last 7 days, what was the SEVERITY of your SHORTNESS OF BREATH at its WORST?**

- a) None
- b) Mild → Q1b.1: **In the last 7 days, how much did SHORTNESS OF BREATH INTERFERE with your usual or daily activities?**
  Not at all / A little bit / Somewhat / Quite a bit / Very much

  Q1b.2: **Is/was this symptom new or worsening?**
  - Yes → Q1.e2.1: **Have you discussed this change with your clinical team?**
    - Yes
    - No
  - No

  Q1b.3: **Please describe your degree of breathlessness related to activities.**
  1) Only during strenuous exercise
  2) After climbing a flight of stairs
  3) After walking just a few minutes on level ground
  4) I'm breathless engaging in daily activities such as undressing
  5) At rest

*Added for constipation, nausea, vomiting, shortness of breath, swelling

This is a Multimedia Appendix to a full manuscript published in the J Med Internet Res. For full copyright and citation information see http://dx.doi.org/10.2196/jmir.18982
